# Plasticity, injury-induced reprogramming, and translational applications of Schwann cells in neural regeneration

**DOI:** 10.3389/fncel.2026.1845736

**Published:** 2026-06-08

**Authors:** Ye Zhu, Jingfei Zhong, Zhongpeng Wang, Xiaosong Gu, Shanshan Wang, Meiyuan Li

**Affiliations:** 1 Academy of Medical Engineering and Translational Medicine, Tianjin University, Tianjin, China; 2 Key Laboratory of Neuroregeneration of Jiangsu and Ministry of Education, Co-innovation Center of Neuroregeneration, NMPA Key Laboratory for Research and Evaluation of Tissue Engineering Technology Products, Nantong University, Nantong, Jiangsu, China; 3 Nantong University Xinglin College, Nantong, Jiangsu, China; 4 Department of Obstetrics and Gynecology, Affiliated Hospital of Nantong University, Nantong, Jiangsu, China

**Keywords:** myelination, neural regeneration, peripheral nerve injury, Schwann cell, spinal cord injury

## Abstract

Schwann cells (SCs), the predominant glial cell population in the peripheral nervous system (PNS), have undergone a paradigm shift from historically passive structural components of myelinated axons to active, multifunctional regulators of neural development, regeneration, and neuropathology. This review briefly outlines Schwann cell developmental origin as a biological backdrop, while centering on their inherent phenotypic plasticity and translational applications. Following peripheral nerve injury, SCs rapidly undergo context-dependent dedifferentiation and transcriptional reprogramming, acquiring a regenerative phenotype characterized by phagocytic activity, secretion of neurotrophic factors, and structural reorganization into Büngner bands. Notably, both endogenous and exogenously delivered SCs demonstrate capacity to migrate into lesioned central nervous system (CNS), including spinal cord injury sites, where they contribute to remyelination, modulation of glial scar formation, and partial restoration of electrophysiological connectivity and behavioral function. These attributes collectively establish SCs as phenotypically adaptable cellular mediators capable of facilitating neural repair across anatomically and functionally distinct compartments. To inform translational efforts, this review critically evaluates emerging strategies, including autologous cell transplantation and SC-derived exosomes, by appraising their mechanisms, limitations, and future perspectives. This review aims to deepen the mechanistic understanding of Schwann cell biology and provide a theoretical basis for the development of regenerative treatments for peripheral nerve injury and spinal cord injury.

## 1 Introduction

SCs have long been recognized as the principal glial cells of the PNS, primarily appreciated for their role in forming myelin sheaths that enable rapid saltatory conduction. For much of the past century, this structural function defined their identity in neurobiology. However, a growing body of evidence over recent decades has fundamentally reshaped this view, revealing SCs as remarkably versatile cellular entities that actively orchestrate neural development, respond dynamically to injury, and even extend their reparative influence beyond the confines of the PNS.

The developmental journey of SCs is itself a testament to their complexity (Stundl et al., 2025). Originating from multipotent neural crest cells (NCCs), they traverse a precisely choreographed sequence of differentiation—from Schwann cell precursors (SCPs) to immature SCs (iSCs), and ultimately to mature myelinating or non-myelinating subtypes. This progression is governed by an intricate network of signaling pathways, including NRG1/ErbB, Notch, and Wnt/β-catenin, which coordinate critical events such as radial sorting and subtype specification (Saito et al., 2012; Soós et al., 2025). Yet, what truly distinguishes SCs from other glial populations is their extraordinary plasticity. Unlike oligodendrocytes in the CNS, mature SCs retain the capacity to reversibly alter their differentiation state in response to environmental cues (Weiss et al., 2021). Following peripheral nerve injury (PNI), they undergo rapid dedifferentiation and transcriptional reprogramming, acquiring a repair phenotype that enables them to clear myelin debris, secrete neurotrophic factors, recruit immune cells, and form Büngner bands—specialized cellular scaffolds that guide regenerating axons toward their targets (Jessen and Arthur-Farraj, 2019).

Intriguingly, the regenerative capabilities of SCs are not confined to their native PNS environment (Li et al., 1999; Lu et al., 2025). Following spinal cord injury (SCI), both endogenous SCs and those delivered exogenously have been shown to infiltrate the lesion site, where they contribute to remyelination, modulate glial scar formation, and facilitate partial functional recovery. This cross-compartmental reparative capacity has positioned SCs as uniquely attractive candidates for CNS repair strategies, spurring the development of therapeutic strategies ranging from autologous cell transplantation to cell-free interventions utilizing SC-derived exosomes (SC-exos) (Wang et al., 2024; Andriot et al., 2025).

Owing to their intrinsic phenotypic plasticity, SCs actively engage in a spectrum of non-neural pathological processes—encompassing inflammatory regulation, tissue repair, fibrotic remodeling, and stromal modulation in tumor microenvironments (Parfejevs et al., 2018; Deborde et al., 2022; Wu et al., 2023). These newly recognized roles challenge the traditional view of SCs as lineage-restricted peripheral glia and highlight their broader pathophysiological relevance across multiple disease domains. Within the nervous system, however, the present review focuses on SC plasticity, PNI, and spinal cord repair, and acknowledges that the principles discussed may extend to broader contexts.

Despite substantial advances in our understanding of SC biology, a comprehensive review that bridges their developmental regulation, injury-induced plasticity, and emerging therapeutic applications remains lacking. Existing literature often addressed these facets in isolation, obscuring the unifying principles that govern SC behavior across contexts. This review aims to fill this gap by integrating these interconnected dimensions. After briefly outlining SC development, we then explore the plasticity that enables their functional reprogramming following PNI. Finally, we critically evaluate the therapeutic landscape for SCI, comparing cell-based and exosome-based strategies with respect to their mechanistic foundations, current limitations, and emerging innovation trajectories (Fan et al., 2025).

By synthesizing these perspectives, we seek to provide a mechanistic framework that informs both fundamental understanding and the rational design of next-generation regenerative therapies for traumatic injuries affecting the nervous system.

## 2 Development of Schwann cells

SCs arise from multipotent NCCs via a well-defined, four-stage developmental cascade: (1) migratory NCCs, (2) SC precursors SCPs, (3) iSCs, and (4) terminally differentiated myelinating SCs (mSCs) or non-myelinating SCs (nmSCs) (Stavely et al., 2022; Stierli and Sommer, 2022; Kantarci et al., 2024; Stundl et al., 2025). NCCs undergo epithelial-to-mesenchymal transition and delaminate from the dorsal neural tube in response to coordinated bone morphogenetic proteins (BMP), fibroblast growth factors (FGFs), and Wnt signaling (Saito et al., 2012; Soós et al., 2025). Subsequently, SCP specification is governed by the transcription factor Sox10 and the neuregulin-1 (NRG1)/ErbB receptor tyrosine kinase signaling axis (Woodhoo and Sommer, 2008; Tseropoulos et al., 2024). SCPs proliferate and differentiate into iSCs, which engage in radial sorting—a morphogenetic process wherein SCs selectively associate with axons according to caliber, thereby establishing one-to-one pre-myelinating relationships (Doan and Monk, 2025). Finally, iSCs commit to one of two mutually exclusive differentiation fates. In the myelinating lineage, SCs spiral-ensheath large-diameter axons and activate a transcriptional program centrally regulated by the zinc-finger protein Krox20 (Egr2) (Pestronk et al., 2023; Wilson et al., 2024). In the non-myelinating lineage, SCs collectively ensheath small-diameter axons to form Remak bundles—a fate actively maintained by low-level NRG1 type III signaling and sustained Notch pathway activity (Woodhoo et al., 2009; Wilson et al., 2024; Pardo-Rodríguez et al., 2025). These stage-specific transitions are tightly coordinated by multiple intersecting signaling pathways. Table 1 provides a systematic overview of the principal regulatory pathways/transcription factors, specifying their temporal windows of activity and predominant functional roles during SC development.

**Table 1:** Core signaling pathways and transcription factors regulating SC lineage development.

Core signaling pathway/Transcription factor	NCC specification and migration	NCC-to-SCP specification	SCP-to-iSC differentiation	SC maturation	References
Wnt/β-Catenin	Induce NCC specification and boundary establishment	Promote SCP proliferation; Inhibit premature differentiation	Regulate axonal radial sorting	—	Saito et al. (2012), Grigoryan et al. (2013), Soós et al. (2025), and Zhang Q. Y. et al. (2025)
BMP	Induce NCC fate; Inhibit neural lineage differentiation	—	—	—	Gross et al. (1996), Jessen and Mirsky (2008), and Han et al. (2026)
FGF	Promote NCC specification and proliferation; Maintain NCC stemness	—	—	—	Martinez-Morales et al. (2011) and Han et al. (2026)
Hedgehog (Shh/Dhh)	Restrict NCC neural lineage fate; Establish dorsoventral boundary	Promote SCP survival and proliferation	Facilitate iSC differentiation; Initiate pre-myelination program	Drive perineurial sheath formation; Reinforce core myelin gene expression	Parmantier et al. (1999), Zhang Q. et al. (2025), and Han et al. (2026)
Notch	Maintain NCC stemness; Bias toward glial lineage	Maintain SCP immature state	Continuously inhibit myelination initiation	Relieve inhibition of differentiation	Woodhoo et al. (2009), Wu et al. (2016), Piovesana et al. (2022), and Dai et al. (2025)
Sox10	Master switch of SC lineage fate	Cooperate to lock in SC fate	Continuously maintain lineage identity	Cooperate with Krox20 to activate core myelin genes	Finzsch et al. (2010), Lai et al. (2021), and Podder et al. (2025)
NRG1/ErbB	—	Ensure SCP survival, migration and proliferation	Initiate pre-myelination program	Drive myelin growth, terminal differentiation and thickness regulation	Glenn and Talbot (2013), Huang et al. (2023), and Tseropoulos et al. (2024)
HDACs	—	Epigenetically activate SC-specific genes	—	—	Jacob et al. (2014), Tseropoulos et al. (2024), and Yu et al. (2024)
cAMP/PKA	—	—	Drive SCP-to-iSC conversion and acquisition of survival autonomy	Activate Oct6-Krox20 transcriptional cascade	Glenn and Talbot (2013) and Wilson et al. (2024)
Hippo/YAP	—	—	Regulate iSC proliferation-differentiation balance	—	Poitelon et al. (2016) and Tseropoulos et al. (2024)
TGF-β	—	—	—	Inhibit myelination; Promote nmSC differentiation	Einheber et al. (1995) and Ou et al. (2022)

—: No clearly reported major functions or relatively low activity at this developmental stage.

## 3 Plasticity of Schwann cells

Unlike oligodendrocytes in the CNS, mature mSCs retain substantial intrinsic phenotypic plasticity—defined as the capacity to reversibly transition between differentiation states in response to microenvironmental signals (Weiss et al., 2021). In demyelinating peripheral neuropathies and peripheral nerve tumors, SCs undergo pathological dedifferentiation accompanied by aberrant proliferation, thereby increasing their susceptibility to autoimmune-mediated damage or oncogenic transformation (Renthal et al., 2020). Conversely, under conditions of PNI, this same plasticity enables functional reprogramming of SCs into a repair phenotype—a process that is essential for effective axonal regeneration (Dai et al., 2025; Ren et al., 2025). Thus SC plasticity plays a dual role by facilitating regenerative processes after PNI while potentially promoting disease pathogenesis under conditions of dysregulation.

Following PNI, denervated SCs rapidly downregulate myelin-associated genes including Krox20, MBP, and P0—while concurrently upregulating repair-associated transcription factors and receptors such as c-Jun, p75NTR, and Sox2 (Liu et al., 2019; Wilcox et al., 2020; Deininger et al., 2024). The transcription factor c-Jun is widely established as a master regulator of the SC repair program: conditional ablation of c-Jun in SCs severely compromises axonal regeneration, whereas sustained c-Jun activation robustly induces the expression of multiple repair-related effectors (Arthur-Farraj et al., 2012; Wagstaff et al., 2021; Xu X. et al., 2023). Subsequently, SCs reorganize into longitudinally aligned columns within their native basal lamina tubes, forming Büngner bands—specialized cellular structures that provide both physical guidance channels and localized molecular cues essential for directed axonal regrowth (Jessen and Arthur-Farraj, 2019; Wang et al., 2023).

The SC repair response is not homogeneous but instead exhibits substantial spatial and temporal heterogeneity. Single-cell and single-nucleus transcriptomic analyses have revealed functionally specialized subpopulations shaped by injury type, anatomical location, and intercellular communication (Ou et al., 2022; Yim et al., 2022; Heming et al., 2025). At nerve transection sites, direct contact with fibroblasts drives SC migration through integrin and cytokine dependent signaling cascades (Zhang et al., 2016; Li et al., 2022; Li et al., 2023). Macrophages recruited to lesion sites also modulate SC plasticity via paracrine factors, establishing a reciprocal signaling circuit that amplifies the endogenous repair cascade (Cattin et al., 2015; Li et al., 2025).

An emerging consensus holds that the repair SC phenotype does not represent a mere reversion to an immature developmental state, but rather constitutes a distinct, injury-induced cellular reprogramming event—functionally and molecularly tailored to the local microenvironment. Partial reprogramming of senescent SCs has been shown to restore stress granule homeostasis, reverse hallmarks of cellular senescence, and significantly improve peripheral nerve regeneration in aged animal models (Wang et al., 2025).

Despite substantial progress, critical conceptual challenges and unresolved controversies persist concerning several foundational aspects of SC plasticity. First, no consensus molecular definition exists for repair SC phenotype. Studies variably define it based on elevated c-Jun activity alone or through combinatorial expression of additional markers including p75NTR, Sox2, and Gap43, reflecting divergent operational criteria across experimental systems (Ebenezer et al., 2011; Arthur-Farraj et al., 2012; Suzuki et al., 2023). Second, it remains unclear whether denervated SCs undergo uniform reprogramming into the repair phenotype or whether only a spatially and temporally restricted subpopulation acquires this identity. Moreover, whether they fully redifferentiate into mature myelinating cells or instead adopt a stable non-myelinating phenotype—is still incompletely resolved. Third, single-cell transcriptomic and epigenomic profiling has revealed profound functional heterogeneity within the repair SCs. This has led to the hypothesis that the canonical repair phenotype actually comprises multiple lineage related subpopulations, each specialized for discrete regenerative functions such as axon guidance, immunomodulatory signaling and myelin debris clearance.

Resolving these unresolved questions will require integrated experimental strategies that combine cell-type-specific genetic lineage tracing with longitudinal, single-cell multi-omic profiling. Such approaches will not only reveal the mechanistic underpinnings of SC plasticity but also expose critical knowledge gaps demanding rigorous functional validation. Deciphering the molecular determinants that establish and maintain repair SC identity and drive their functional diversification will enable rational design of targeted interventions to harness this intrinsic plasticity for regenerative therapies in both PNS and CNS disorders.

## 4 Response of Schwann cells following peripheral nerve injury

During development, SCs follow a well-defined differentiation program to establish myelinated and non-myelinated nerve fibers essential for normal peripheral nerve function. However, following PNI, this homeostatic identity is rapidly disrupted. SCs lose their mature phenotypes, undergo dramatic dedifferentiation, and exhibit remarkable cellular plasticity and heterogeneity (Nie et al., 2024; Qian et al., 2024). Instead of maintaining myelin sheaths or supporting axons in a steady state, they re-enter the cell cycle, migrate to the injury site, clear myelin debris, secrete neurotrophic factors, and form organized cellular tracks to guide regenerating axons (Wang et al., 2021; Zhang H. M. et al., 2025; Mironova et al., 2026). This profound phenotypic switch not only highlights the extraordinary adaptability of SCs but also underscores their central role in determining the success or failure of peripheral nerve regeneration.

In preclinical PNI research, nerve crush and nerve transection stand as two widely used canonical experimental models. Nerve crush induces axonotmesis—a lesion in which axons degenerate while the endoneurial tubes (comprising the basal lamina, connective tissue sheaths, and associated SCs) remain anatomically preserved (Chadwick et al., 2025). Consequently, rodents subjected to this model exhibit rapid and robust axonal regeneration, with functional recovery typically achieved within 3–4 weeks. In contrast, nerve transection produces neurotmesis, characterized by complete disruption of axons, connective tissue architecture, and basal lamina integrity (Zhai et al., 2026). Under such conditions, surgical intervention (such as conduit implantation or direct epineurial repair) is required to re-establish continuity between the proximal and distal nerve stumps. This intervention creates a confined microenvironment that facilitates intimate axon-SCs interactions and supports coordinated regrowth toward the distal target (Han et al., 2024). A single-cell sequencing analysis conducted at various time points following sciatic nerve transection injury in rats delineated distinct subtypes of SCs. Specifically, partially myelinating SCs were present at the lesion site during the acute phase (0 day), yet a universal deficiency of all SCs subtypes was observed at the lesion site prior to day 5. Subsequently, functionally distinct populations including remyelination, proliferating, and repair-associated SCs (Gli0-Gli5) progressively emerged, exhibiting increasing heterogeneity over time (Ouyang et al., 2025).

Although axonal degeneration in the distal stump begins 2–4 days post-injury, SCs initiate their response to axonal disruption much earlier—typically within hours (Gargareta et al., 2024). This rapid activation suggests that damaged axons actively signal SCs to initiate a coordinated response to axonal degeneration, likely via conserved molecular cues (Gupta et al., 2023). Subsequently, SCs undergo a well-orchestrated phenotypic transition includings dedifferentiation and activation, followed by proliferation and functional reprogramming to support regeneration.

Prior to myelination, SCs transiently upregulate characteristic markers of the immature SC stage, including c-Jun, p75NTR and Sox2, while concurrently downregulating myelin-associated genes (Chun et al., 2022). These include the master transcriptional regulator Krox20, enzymes essential for myelin lipid biosynthesis (e.g., cholesterol synthase), and structural myelin proteins such as MBP, P0, and periaxin (Jha et al., 2020; Huang G. J. et al., 2024; Su et al., 2025).

Subsequently, SCs acquire an emerging, injury-specific phenotype, which is distinct from both quiescent SCs in intact nerves and developmentally immature SCs (Li et al., 2025). These phenotypic adaptations collectively support nerve repair through three interdependent functional modules: (1) neurotrophic support, (2) immune modulation, and (3) myelin clearance and structural reorganization.

First, SCs upregulate a defined set of neurotrophic factors—including glial cell line-derived neurotrophic factor (GDNF), brain-derived neurotrophic factor (BDNF), neurotrophin-3 (NT-3), nerve growth factor (NGF), vascular endothelial growth factor (VEGF), and leukemia inhibitory factor (LIF)—which enhance neuronal survival and stimulate axonal elongation (Zhang et al., 2020; Proietti et al., 2021; Baraz et al., 2026). Second, SCs at the distal stump secrete immunomodulatory cytokines such as tumor necrosis factor (TNF)-α, interleukin (IL)-1α, IL-6, and monocyte chemoattractant protein-1 (MCP-1)—to recruit and activate macrophages (Wei et al., 2025). Third, SCs initiate intrinsic myelin degradation, primarily via autophagy and calpain-mediated proteolysis, accounting for the majority of myelin breakdown within 5–7 days post-injury (Patel et al., 2024). Critically, these processes are functionally integrated. Beyond macrophage recruitment, cytokines such as IL-6 directly act on injured neurons to facilitate axon regeneration. Infiltrating macrophages amplify this signaling cascade by secreting additional cytokines and growth factors, and cooperatively clear inhibitory myelin debris together with SCs, while promoting angiogenesis within the distal nerve stump (Wei et al., 2022; Kalpachidou et al., 2025). Finally, SCs undergo morphological and organizational remodeling to form regeneration tracks. Former myelinating SCs dedifferentiate, acquire a slender bipolar shape and arrange longitudinally inside inherent basal lamina tubes to construct Büngner bands (Huang Y. Y. et al., 2024; Wang et al., 2025). To elaborate, we have comprehensively characterized the morphological and functional alterations of SCs across distinct stages of this process. Notably, SCs exhibit substantial heterogeneity in critical molecular events, gene expression dynamics, and activation patterns of key signaling pathways (Table 2).

**Table 2:** Temporal changes in SCs following peripheral nerve injury.

Pathological process	Key events	Molecular and cellular changes	Signaling pathways
Detachment and Initial Stress Response	Axon-glia junction disruption Myelin sheath loosening and retraction Loss of maintenance signals	Upregulated/Activated: Proteases (Calpain, Caspase-3) Intracellular Ca^2+^ elevation Downregulated/lnactivated: Myelin proteins (MBP, P0) Axonal signals (NRG1/ErbB)	NRG1/ErbB signaling downregulation Calcium overload, calpain activation Integrin signaling reorganization
Dedifferentiation Initiation	Phenotypic switch from “maintenance” to “repair” mode Cessation of myelin protein synthesis Activation of phagocytosis-related genes	Upregulated: TFs (c-Jun, JunD) Repair markers (p75NTR, GFAP, L1CAM) Autophagy (LC3) Downregulated: Myelination TFs (Krox20, Oct6) Myelin proteins (MBP, P0, PMP22)	JNK/c-Jun axis (core driver) p38 MAPK (inflammatory stress) Autophagy induction
Active Dedifferentiation and Proliferation	Extensive myelinolysis Massive SC proliferation Formation of SC columns	Upregulated: Proliferation markers (Ki67, PCNA, Cyclin D1) Phagocytic receptors (MEGF10, GULP1) Lysosomal enzyme (Cathepsin D) Inflammatory cytokines (MCP-1, IL-6, TNF-α) Downregulated: Myelination TFs (Krox20, Oct6) Myelin proteins (MBP, P0, PMP22)	ERK/MAPK (proliferation) NF-κB (cytokine transcription) Rac1/Rho GTPases (cytoskeleton)
Clearance and Macrophage Cooperation	Collaborative debris clearance with macrophages Mature Büngner band formation Secretion of neurotrophic factors	Upregulated: Chemokines (MCP-1, CXCL1) Neurotrophic factors (BDNF, GDNF, NGF) ECM components (Laminin, tenascin-C) Sustained high expression: Repair markers (c-Jun, p75NTR, GFAP)	JAK/STAT3 (neurotrophic effects) cAMP/PKA (repair phenotype) PPARγ (lipid metabolism) mTORC1 suppression
Repair Phenotype Maintenance	Maintenance of Büngner band structure Secretion of axon guidance molecules Waiting for axonal sprouting	Sustained expression: Repair markers (c-Jun, p75NTR, GFAP) ECM receptors (Integrin α7β1) Downregulated: Inflammatory cytokines (MCP-1, CXCL1, IL-6, TNF-α)	Integrin-ECM signaling (survival) PI3K/Akt (survival) RhoA/ROCK (cell polarity)
Axonal Recontact & Remyelination	Axonal recontact with SCs Redifferentiation into myelinating phenotype Initiation of remyelination	Upregulated/Re-expressed: Myelination TFs (Krox20, Oct6) Myelin proteins (MBP, P0, PMP22) Cell cycle exit marker (p27^kip1^) Downregulated: Repair markers (c-Jun, p75NTR, GFAP)	NRG1/ErbB reactivation mTORC1 activation (myelin synthesis) Notch signaling downregulation

Although SCs re-enter the cell cycle after injury and expand several-fold at the distal stump, peripheral nerve regeneration after crush injury remains equally effective in mice with inhibited SC proliferation, demonstrating that the transition of myelinating SCs to repair SCs is independent of cell cycle progression (Ye et al., 2023; Huang Y. Y. et al., 2024).

## 5 Role of Schwann cells in spinal cord injury

It has been confirmed that endogenous SCs can invade and migrate to the injured spinal cord (Li et al., 1999; Lu et al., 2025). For example, in spinal cords with contusion or photochemical damage, SCs are detectable at injury sites alongside regenerating axons (Kromer and Cornbrooks, 1985). Early studies also revealed that regenerating axons have myelin or a large number of SCs invading sheaths, as well as bundles where axonal SC units are embedded in a dense ECM, at least partially derived from ependymal cells of the central canal proliferation zone (Zheng and Tuszynski, 2023; Kühne et al., 2026).

### 5.1 Mechanisms of Schwann cells in spinal cord injury repair

This observation seems to contradict conventional wisdom, as SCs are primarily myelin-producing cells of the PNS. Therefore, what is the underlying reason for the beneficial effects of SCs on SCI repair? As a matter of fact, the healthy CNS is not conducive to the survival of SCs. However, once SCI occurs, the blood-spinal cord barrier is disrupted, the local microenvironment undergoes significant changes, and a large number of chemotactic factors are released to recruit SCs. These changes attract SCs to migrate in and enable them to survive. Once they enter the damaged area, SCs exhibit three major capabilities that are critical for PNS repair: (1) strong regenerative capacity via secretion of neurotrophic factors; (2) efficient phagocytic function to clear inhibitory myelin debris; and (3) excellent myelination capacity to restore saltatory conduction. In the context of SCI, SCs exert their recruitment, survival, and reparative functions through an interconnected molecular signaling network. Initially, the chemokine (CXCL12/CXCR4) axis together with damage-associated molecular patterns guides the migration of SCs toward the lesion core (Chu et al., 2017; Negro et al., 2017). After infiltration, NRG1/ErbB signaling and the transcription factor Sox10 collectively sustain the repair SC phenotype (Yamauchi et al., 2008; Huang et al., 2023; Podder et al., 2025). The upregulation of c-Jun further initiates the core regenerative program, driving the expression of neurotrophic factors (BDNF, NGF, GDNF) and matrix metalloproteinases (MMPs) (Yang et al., 2020; Wan et al., 2024). These factors provide trophic support for damaged axons, whereas MMPs degrade inhibitory chondroitin sulfate proteoglycans, and adhesion molecules contribute to a permissive growth substrate. Meanwhile, the PI3K/Akt and MAPK/ERK signaling pathways facilitate SC survival and proliferation, whereas STAT3 signaling promotes the secretion of anti-inflammatory IL-10, thereby inducing macrophage M2 polarization and remodeling the local inflammatory microenvironment toward a pro-repair profile (Ishii et al., 2021; Ren et al., 2023; Yu et al., 2024; Zhang H. M. et al., 2025). When traversing the astrocyte-derived glial scar, SCs interact with ECM via integrins and modulate boundary interplay with reactive astrocytes through Eph/ephrin signaling (Afshari et al., 2010; Garcia-Diaz et al., 2019). Additionally, SCs secrete VEGF to promote angiogenesis and improve oxygen supply and tissue perfusion (Huang et al., 2022). Ultimately, the cAMP/PKA pathway upregulates Krox20 and triggers the expression of myelin proteins MBP, and P0, enabling efficient remyelination of denuded axons and restoring neural conduction velocity (Woodhoo et al., 2009; Liu et al., 2019; Wilcox et al., 2020; Liu et al., 2023). Collectively, this synergistic molecular network constitutes an integrated mechanism underlying SC-mediated spinal cord repair. More specifically, Table 3 summarizes the key mechanisms and representative molecules of SCs involved in the repair process of SCI.

**Table 3:** Functional category and underlying molecular mechanisms of SCs in spinal cord injury repair.

Functional category	Key molecules	Signaling pathway/Regulatory processes	Specific mechanisms	References
Migration & Invasion	Dock7, Rac1, Cdc42, CXCL12/CXCR4, MMP2/9	MAPK/ERK; Rho GTPase	Disrupt the glial limitans after SCI; Recruit SCs via chemokine CXCL12/CXCR4 signaling; Mediate ECM degradation and cell migration via cytoskeletal regulators and MMPs.	Sims et al. (1998), Yamauchi et al. (2008), Chu et al. (2017), Negro et al. (2017), and Muscella et al. (2020)
Survival & Adaptation	PTEN, Akt, Bcl-2, p75NTR, TrkB, mTOR	PI3K-Akt; Neurotrophic Factor Signaling	Alleviate inhibitory microenvironment following blood–spinal cord barrier disruption; Bind neurotrophic factors to receptors and inhibit apoptosis; Enhance cell survival through PI3K-Akt–mTOR pathway.	Liu et al. (2019), Wilcox et al. (2020), Ishii et al. (2021), and Deininger et al. (2024)
Axon Regeneration Promotion	BDNF, NGF, GDNF, c-Jun, Sox2, ATF3	MAPK/ERK; Transcriptional Regulation	Secrete neurotrophic factors to support axon growth; Drive dedifferentiation and enhance plasticity via transcription factors; Form Büngner band-like structures to guide regenerating axons.	Yang et al. (2020), Huang et al. (2022), and Wan et al. (2024)
Myelin Debris Clearance	TAM Receptors (Tyro3, Axl, Mer), LC3, Beclin1	Autophagy Signaling; Phagocytosis	Phagocytose myelin debris via TAM receptor-mediated phagocytosis; Degrade internalized debris through autophagy; Eliminate axon regeneration inhibitors from the injury site.	Brosius Lutz et al. (2017), Li et al. (2020), Wan et al. (2021), and Gambarotto et al. (2025)
Remyelination	Krox20, Sox10, Oct6, MBP, P0	Myelin Gene Transcription	Redifferentiate SCs to restore myelination function; Promote myelin gene expression via transcription factors; Wrap regenerated axons to form new myelin sheaths.	Woodhoo et al. (2009), Liu et al. (2019), Wilcox et al. (2020), and Liu et al. (2023)
Inflammatory Regulation	TNF-α, IL-1β, TGF-β, HDACs	Inflammatory Signaling; Epigenetic Regulation	Inhibit pro-inflammatory factor release; Regulate chromatin remodeling via HDACs; Promote microglia polarization to anti-inflammatory phenotype.	Einheber et al. (1995), Yadav et al. (2021), Ou et al. (2022), and Wang et al. (2024)

Although SCs only remyelinate approximately 5% of surviving sensory axons, this limited remyelination is sufficient to restore functional action potential conduction (Yum et al., 2024). Importantly, the invasion of endogenous SCs into the injured spinal cord is not restricted to experimental models (Lu et al., 2025). In human patients with chronic SCI, abundant SCs and their direct associations with axons have been consistently observed (Zheng and Tuszynski, 2023). The ability to obtain large quantities of highly purified SCs from the peripheral nerves of animals and humans provides an important foundation for clinical autologous transplantation. In animal models of SCI, transplanted SCs can survive and effectively promote axonal regeneration and remyelination (Lu et al., 2025). Transplanted SCs fail to migrate from the graft site into the host spinal cord, which restricts their ability to enhance axonal growth beyond the implant region (Hajimirzaei et al., 2025). Therefore, inducing sufficient SCs to migrate from nerve roots to the injured spinal cord represents a novel therapeutic approach.

### 5.2 Therapeutic strategies based on Schwann cells for spinal cord injury

**Figure 1:**
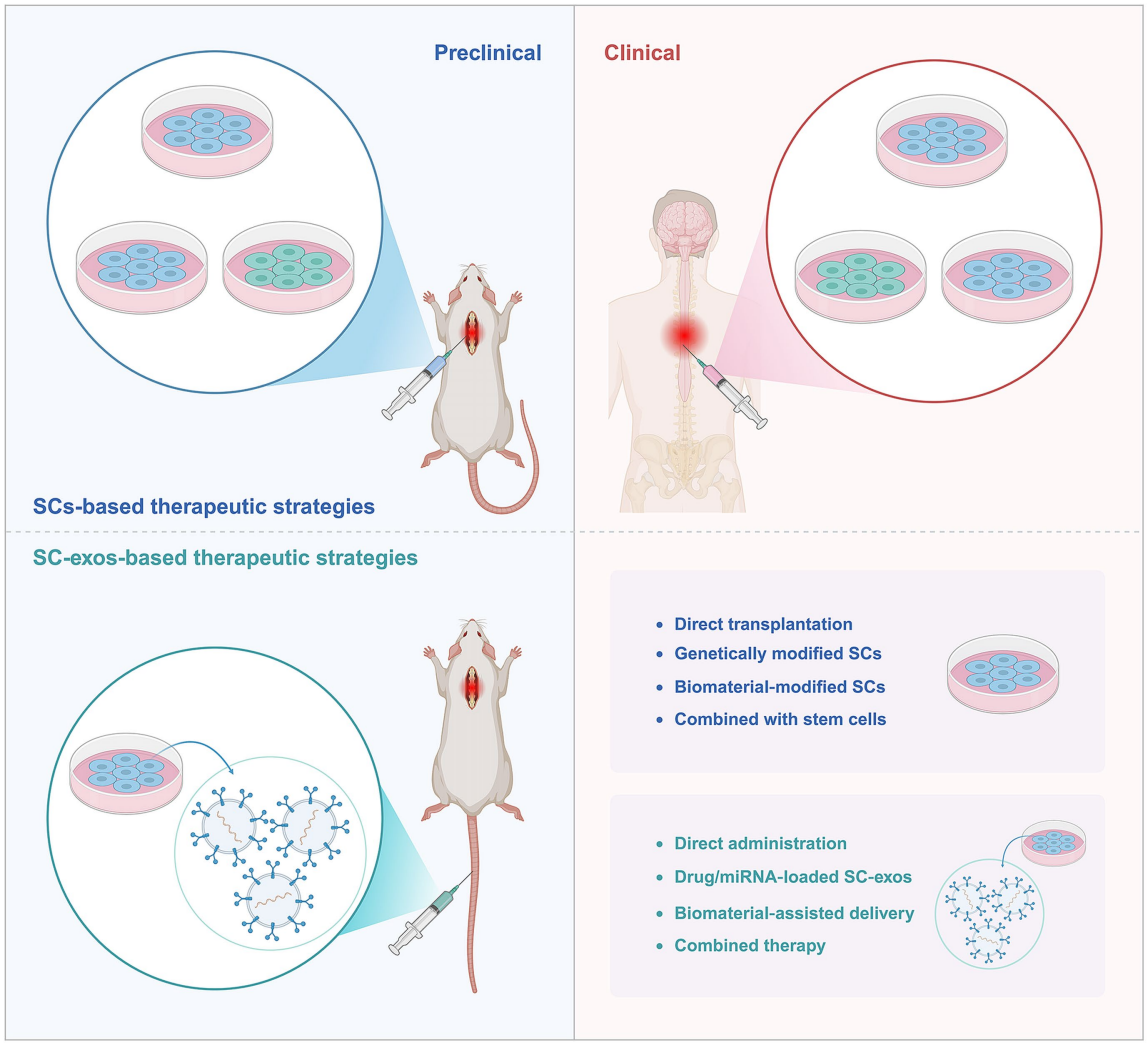
Overview of cell-based and exosome-based therapeutic strategies for spinal cord injury. The schematic summarizes current therapeutic strategies based on SCs for SCI, including the transplantation of SCs alone or in combination in preclinical models (upper left), the injection of SC-exos (lower left), and the transplantation of SCs alone or in combination in clinical trials (upper right); the lower right part outlines the current optimization measures and directions of cell-based/exosome-based therapeutic strategies.

In the current field of SCI repair, Schwann cell-based therapeutic strategies can be broadly divided into cell-based approaches using primary or engineered SCs, and cell-free approaches (Figure 1).

Cell-based strategies include direct intraspinal SC transplantation, genetic or pharmacological modification to enhance their regenerative potential, biomaterial-assisted delivery to promote survival, engraftment, and integration, as well as combination therapy with other stem cell types to exploit synergistic repair effects (Monje et al., 2021; Huang J. S. et al., 2024). Preclinical and clinical evidence indicates distinct therapeutic profiles among SCs, oligodendrocyte precursor cells (OPCs) and mesenchymal stem cells (MSCs). MSCs mainly exert immunomodulatory and neuroprotective effects via paracrine actions; OPCs are specialized in generating central-type myelin, while olfactory ensheathing cells (OECs) display strong migratory capacity to penetrate glial scar (Cao et al., 2010; Granger et al., 2012; Zhou et al., 2016). By contrast, SCs possess unique advantages in migrating into the injured CNS, facilitating axonal regeneration and bridging lesion gaps (Assinck et al., 2017; Xu et al., 2022). Quantitative comparisons show that SCs outperform Wharton’s Jelly MSCs in downregulating pro-inflammatory cytokines in SCI models (Kharazinejad et al., 2023). Collectively, each cell type possesses unique inherent strengths and functional limitations, and combinatorial transplantation can integrate their respective advantages while compensating for individual drawbacks, rendering it a promising prospect for future SCI regenerative therapy.

Extracellular vesicles (EVs) are intrinsically heterogeneous, with three canonical natural subtypes including exosomes, microvesicles, and apoptotic bodies. In particular, exosomes derived from SCs (SC-exos) retain the neurotrophic and immunomodulatory properties of parental SCs while avoiding the risks of live cell transplantation, rendering them the most attractive candidate for cell-free SCI therapy (Ngo et al., 2025). For SC-exos-based interventions, strategies include systemic or local administration of native exosomes; bioengineering of exosomes to load therapeutic cargo (e.g., miRNAs, growth factors, or anti-inflammatory agents); incorporation into hydrogels or scaffolds for spatiotemporally controlled release; and co-administration with rehabilitative training, neuromodulation, or immunomodulatory agents to amplify functional outcomes (Huang et al., 2022; Li et al., 2024; Nie et al., 2024).

Given these modalities, combining SC transplantation with other emerging therapeutic strategies may further promote axonal regeneration and functional recovery following SCI (Nie et al., 2024; Wang et al., 2024; Andriot et al., 2025). While SCs-based therapies have advanced extensively into clinical research with demonstrated efficacy in both preclinical models and clinical trials, the therapeutic application of SC-exos is still restricted to preclinical studies, with no human interventional trials reported to date. This review summarizes recent advances in the applications of both cell-based and exosome-based strategies for SCI repair (Table 4).

**Table 4:** Preclinical and clinical applications of Schwann cell-based therapeutic strategies in spinal cord injury.

Therapeutic Source	Animal model/Indication	Treatment strategy	Key findings
Exosomes derived from primary rat SCs	Compressed SCI model	Nanofiber scaffold-integrated hyaluronic acid hydrogel patch for co-delivery of exosomes and methylprednisolone	The composite patch enhanced functional and electrophysiological recovery in SCI rats by promoting M1-to-M2 macrophage polarization, suppressing inflammation, and increasing neuronal survival (Zhu et al., 2023).
Exosomes derived from primary rat SCs	Compressed SCI model	Tail intravenous injection	SC-derived exosomes activated AMPK-mediated mitophagy and reduced oxidative stress, inflammation, and necroptosis (Xu B. et al., 2023).
Exosomes derived from primary rat SCs	Compressed SCI model	Tail intravenous injection	SC-derived exosomes improved locomotor function in SCI rats as assessed by BBB score, electrophysiology, and CatWalk XT gait analysis (Ren et al., 2023).
SCs from autologous sural nerve	Chronic SCI	Intralesional cell administration	One participant experienced a 4-point improvement in motor function, a 6-point improvement in sensory function, and a 1-level improvement in neurological injury level (Gant et al., 2022).
SCs from autologous sural nerve	Complete SCI	Intrathecal cell administration (combined with BMSCs)	The treatment group showed significantly higher total and domain scores of incontinence quality of life than the control group at 6 months (Akhlaghpasand et al., 2025a).
SCs from autologous sural nerve	Complete SCI	Intrathecal cell administration (combined with BMSCs)	Significant improvements in ASIA, SCIM-III, and WHOQOL-BREF scores were detected at 12 months post-treatment (Akhlaghpasand et al., 2025b).

**Figure 2:**
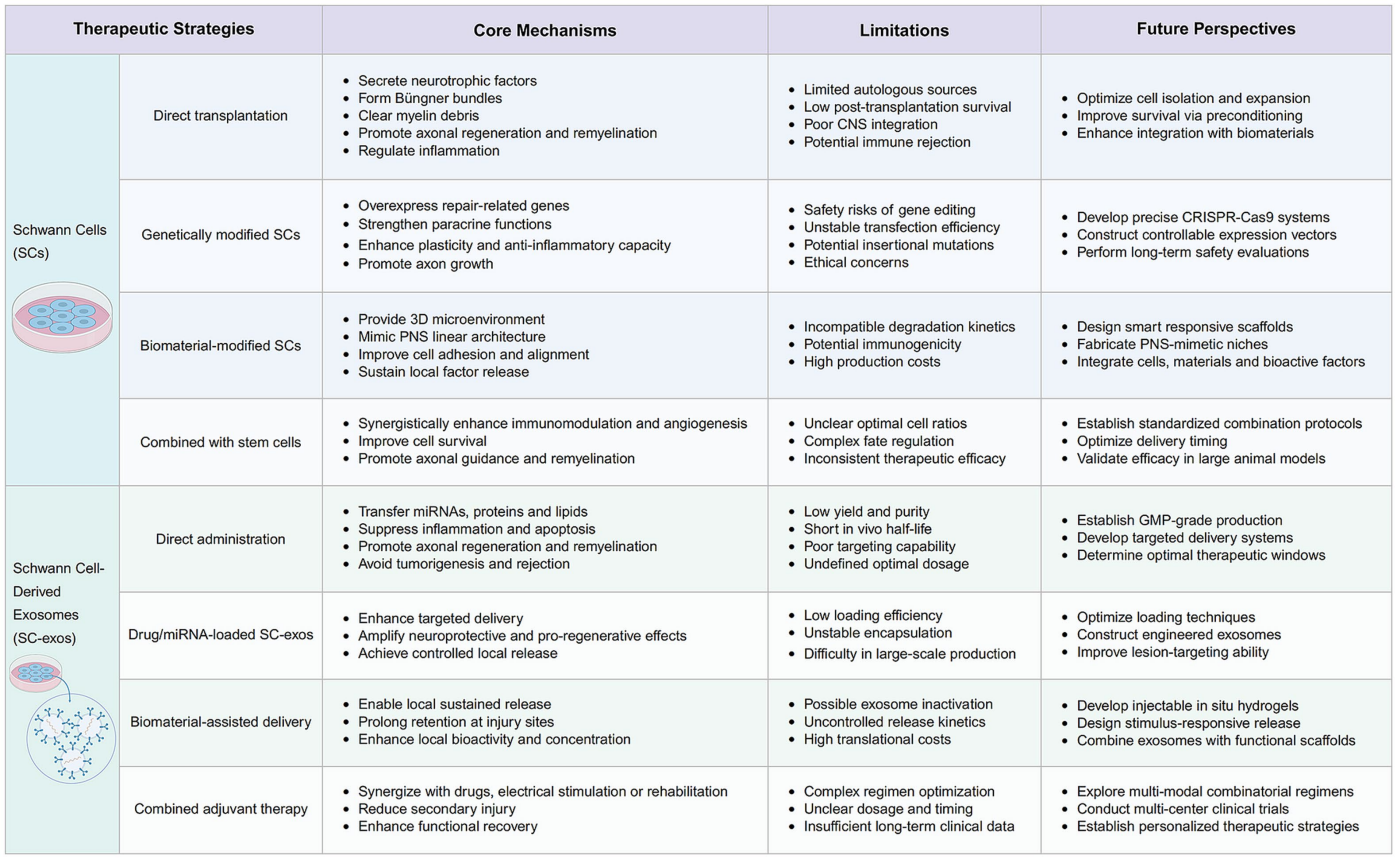
Graphical table summarizing the mechanisms, limitations, and future perspectives of cell-based/exosome-based therapeutic strategies for spinal cord injury. The information is presented in color-zoned modular layout to facilitate direct comparison across multiple therapeutic strategies.

Collectively, these strategies elicit multifaceted therapeutic effects—including neuroprotection, suppression of chronic neuroinflammation, promotion of axonal regeneration and elongation, and restoration of myelin integrity—via mechanistically distinct yet partially convergent pathways (Sas et al., 2020; Park et al., 2026). Nevertheless, each modality presents specific translational hurdles related to scalable cell/exosome sourcing, batch-to-batch consistency, *in vivo* stability, biodistribution precision, and regulatory-grade manufacturing standardization (Wu et al., 2026). A comprehensive comparative analysis of these strategies, covering mechanistic foundations, current limitations, and emerging innovation trajectories, is presented in Figure 2, serving as a structured framework to inform evidence-based therapeutic decision-making and accelerate clinical translation.

## 6 Conclusions and future perspectives

SCs have undergone a remarkable conceptual transformation—from passive structural elements of peripheral nerves to dynamic, multifunctional architects of neural development, repair, and disease (Wang et al., 2022). This review has synthesized the molecular logic governing their ontogeny, the extraordinary plasticity that enables their injury-induced reprogramming, and their emerging roles as therapeutic agents for CNS repair. Collectively, the evidence underscores a unifying principle, wherein SCs uniquely reside at the interface of development and regeneration. They integrate diverse environmental cues to coordinate cellular responses across distinct biological contexts. Understanding the molecular logic underlying this phenotypic transition is not only a fundamental biological question but also holds direct translational relevance for traumatic PNI and spinal cord repair.

Despite these advances, several fundamental biological questions remain unanswered. (1) The heterogeneity of repair SCs revealed by single-cell transcriptomics demands functional validation. Distinct SC subpopulations may play specialized roles in axon guidance, phagocytosis, and immunomodulation, and these subpopulations could be selectively mobilized to facilitate neural regeneration. (2) The mechanisms governing SC invasion into injured spinal cord remain poorly understood. Elucidating the chemotactic signals and extracellular matrix remodeling processes that facilitate this cross-compartmental migration may unveil new therapeutic targets for CNS injury. (3) The suboptimal migration of transplanted SCs beyond the graft site limits their ability to support axonal growth over extended distances—a critical barrier that must be addressed to achieve meaningful functional recovery. The regenerative effect of Schwann cell-based therapeutic strategies is highly contingent upon the environment. The selection of the target, the timing of modification, the maturity stage of the cells, and the microenvironment of transplantation are all crucial factors that determine the success of the treatment.

Beyond these unresolved biological concerns, several translational obstacles demand explicit and rigorous attention for future clinical translation. (1) Scalability and GMP compliance: Autologous SC isolation requires invasive nerve biopsy, inevitably causing donor-site morbidity, whereas allogeneic SCs carry inherent immunogenic risks. (2) Cell variability: SCs harvested from different donors or cultured under divergent conditions exhibit variable expression of repair-related markers, resulting in inconsistent therapeutic efficacy across batches. (3) Delivery efficiency: Following intraspinal injection, grafted cells exhibit poor early survival and limited migratory capacity, remaining confined to the vicinity of the injection site. Such biological constraints greatly hinder large-scale axonal regeneration and lesion gap repair. (4) Long-term safety: Although tumorigenic risk remains low, undesirable sensory abnormalities such as allodynia have been documented in SC transplantation models; additionally, the long-term biosafety profiles of SC-exos remain largely uncharacterized. Addressing these multidimensional challenges will be central to advancing SC-based therapies toward clinical application.

Looking forward, emerging technological and conceptual innovations offer pathways to overcome these limitations. Advances in single-cell and spatial transcriptomics will enable precise mapping of SC heterogeneity across injury contexts, informing the design of targeted interventions. The intersection of SC biology with mechanobiology and aging research, particularly the recent finding that partial reprogramming reverses senescence in aged SCs, opens new avenues for rejuvenating repair capacity in older populations (Fuentes-Flores et al., 2023). Furthermore, the convergence of SC-based therapies with biomaterial engineering holds significant promise. Smart hydrogels that mimic the native extracellular matrix, deliver sustained release of neurotrophic factors, or provide topographical guidance cues could substantially enhance the efficacy of transplanted SCs. Beyond naturally secreted exosomes, engineered exosomes can be decorated with therapeutic miRNAs, neurotrophic factors, or surface-targeted moieties to achieve improved efficacy and specificity. In parallel, artificially fabricated cytochalasin B-induced membrane vesicles (CIMVs) serve as scalable alternative carriers, expanding the available toolkit of cell-free neuroregenerative therapeutics (Gomzikova et al., 2019; Nair et al., 2021). As major cell-free candidates, SC-exos and other vesicle formats also offer inherent advantages in safety and practical storage, making them ideal platforms for targeted neural repair.

Ultimately, the unique plasticity and regenerative capacity of SCs position them as central players in the future of neural repair. Realizing their full therapeutic potential will require not only deeper mechanistic insight but also creative integration with emerging technologies, from gene editing to tissue engineering, to precisely control SC behavior in space and time. By bridging our understanding of SC plasticity, injury response, and translational applications, this review aims to provide a foundation for the rational design of next-generation interventions for traumatic injuries affecting both the peripheral and central nervous systems.

## References

[ref1] Afshari F. T. Kwok J. C. Fawcett J. W. (2010). Astrocyte-produced ephrins inhibit Schwann cell migration via VAV2 signaling. J. Neurosci. 30, 4246–4255. doi: 10.1523/JNEUROSCI.3351-09.2010, 20335460 PMC6634495

[ref2] Akhlaghpasand M. Tavanaei R. Allameh F. Hosseinpoor M. Toreyhi H. Golmohammadi M. . (2025a). Improvement of neurogenic bladder dysfunction following combined cell therapy with mesenchymal stem cell and Schwann cell in spinal cord injury: a randomized, open-label, phase II clinical trial. World Neurosurg. 194:123402. doi: 10.1016/j.wneu.2024.10.131, 39522809

[ref3] Akhlaghpasand M. Tavanaei R. Hosseinpoor M. Golmohammadi M. Mohammadi I. Jolfayi A. G. . (2025b). Neurological, functional, and quality of life outcomes following combined mesenchymal stem cell and Schwann cell therapy in spinal cord injury: a 9-year experience. Stem Cell Res Ther 16:226. doi: 10.1186/s13287-025-04312-7, 40325467 PMC12054327

[ref5] Andriot T. Ghosh M. Pearse D. D. (2025). Engineered healing: synergistic use of Schwann cells and biomaterials for spinal cord regeneration. Int. J. Mol. Sci. 26:7922. doi: 10.3390/ijms26167922, 40869240 PMC12386486

[ref6] Arthur-Farraj P. J. Latouche M. Wilton D. K. Quintes S. Chabrol E. Banerjee A. . (2012). C-Jun reprograms Schwann cells of injured nerves to generate a repair cell essential for regeneration. Neuron 75, 633–647. doi: 10.1016/j.neuron.2012.06.021, 22920255 PMC3657176

[ref7] Assinck P. Duncan G. J. Hilton B. J. Plemel J. R. Tetzlaff W. (2017). Cell transplantation therapy for spinal cord injury. Nat. Neurosci. 20, 637–647. doi: 10.1038/nn.4541, 28440805

[ref8] Baraz L. S. Ataca E. Oflas N. D. Kosali S. C. Usta B. Oral A. . (2026). Altered NGF and GDNF levels reveal neuroimmune dysregulation in COVID-19 patients. Sci. Rep. 16:9919. doi: 10.1038/s41598-026-40236-9, 41714345 PMC13018267

[ref9] Brosius Lutz A. Chung W. S. Sloan S. A. Carson G. A. Zhou L. Lovelett E. . (2017). Schwann cells use TAM receptor-mediated phagocytosis in addition to autophagy to clear myelin in a mouse model of nerve injury. Proc. Natl. Acad. Sci. USA 114, E8072–E8080. doi: 10.1073/pnas.1710566114, 28874532 PMC5617301

[ref10] Cao Q. He Q. Wang Y. Cheng X. Howard R. M. Zhang Y. . (2010). Transplantation of ciliary neurotrophic factor-expressing adult oligodendrocyte precursor cells promotes remyelination and functional recovery after spinal cord injury. J. Neurosci. 30, 2989–3001. doi: 10.1523/JNEUROSCI.3174-09.2010, 20181596 PMC2836860

[ref11] Cattin A. L. Burden J. J. Van Emmenis L. Mackenzie F. E. Hoving J. J. Garcia Calavia N. . (2015). Macrophage-induced blood vessels guide Schwann cell-mediated regeneration of peripheral nerves. Cell 162, 1127–1139. doi: 10.1016/j.cell.2015.07.021 26279190 PMC4553238

[ref12] Chadwick J. S. Decourt C. Müller F. Maldonado-Lasuncion I. Serger E. Kong G. P. . (2025). Dietary-dependent sensitization of neuronal leptin signaling promotes neural repair after injury via cAMP and gene transcription. Neuron 113, 2839–2855. doi: 10.1016/j.neuron.2025.07.016 40812302

[ref14] Chu T. Shields L. B. E. Zhang Y. P. Feng S. Q. Shields C. B. Cai J. (2017). CXCL12/CXCR4/CXCR7 chemokine Axis in the central nervous system: therapeutic targets for remyelination in demyelinating diseases. Neuroscientist 23, 627–648. doi: 10.1177/1073858416685690, 29283028

[ref15] Chun Y. L. Eom W. J. Lee J. H. Nguyen T. N. C. Park K. H. Chung H. J. . (2022). Investigation of the hydrogen Sulfide Signaling pathway in Schwann cells during peripheral nerve degeneration: multi-omics approaches. Antioxidants 11:1606. doi: 10.3390/antiox11081606, 36009325 PMC9405209

[ref17] Dai W. Y. Miao Y. Yi S. (2025). Schwann cell reprogramming via EMT-like program following peripheral nerve injury and during nerve regeneration. Front. Cell Dev. Biol. 13:1621380. doi: 10.3389/fcell.2025.1621380, 40703656 PMC12283579

[ref18] Deborde S. Gusain L. Powers A. Marcadis A. Yu Y. Chen C. H. . (2022). Reprogrammed Schwann cells organize into dynamic tracks that promote pancreatic Cancer invasion. Cancer Discov. 12, 2454–2473. doi: 10.1158/2159-8290.CD-21-1690, 35881881 PMC9533012

[ref19] Deininger S. Schumacher J. Blechschmidt A. Song J. Klugmann C. Antoniadis G. . (2024). Nerve injury converts Schwann cells in a long-term repair-like state in human neuroma tissue. Exp. Neurol. 382:114981. doi: 10.1016/j.expneurol.2024.114981, 39362479

[ref20] Doan R. A. Monk K. R. (2025). Dock1 functions in Schwann cells to regulate development, maintenance, and repair. J. Cell Biol. 224:e202311041. doi: 10.1083/jcb.202311041, 40105697 PMC11921805

[ref21] Ebenezer G. J. O'Donnell R. Hauer P. Cimino N. P. McArthur J. C. Polydefkis M. (2011). Impaired neurovascular repair in subjects with diabetes following experimental intracutaneous axotomy. Brain 134, 1853–1863. doi: 10.1093/brain/awr086, 21616974 PMC3140859

[ref22] Einheber S. Hannocks M. J. Metz C. N. Rifkin D. B. Salzer J. L. (1995). Transforming growth factor-beta 1 regulates axon/Schwann cell interactions. J. Cell Biol. 129, 443–458. doi: 10.1083/jcb.129.2.443, 7536747 PMC2199906

[ref23] Fan L. Wang T. Y. Liu Y. C. Ma M. R. Liu G. Wang Y. Q. . (2025). Natural polyphenol-functionalized Schwann cell-derived exosomes as a temporal neuromodulation strategy for diabetic periodontitis therapy. ACS Nano 19, 32482–32498. doi: 10.1021/acsnano.5c08885, 40908799

[ref24] Finzsch M. Schreiner S. Kichko T. Reeh P. Tamm E. R. Bosl M. R. . (2010). Sox10 is required for Schwann cell identity and progression beyond the immature Schwann cell stage. J. Cell Biol. 189, 701–712. doi: 10.1083/jcb.200912142, 20457761 PMC2872908

[ref25] Fuentes-Flores A. Geronimo-Olvera C. Girardi K. Necuñir-Ibarra D. Patel S. K. Bons J. . (2023). Senescent Schwann cells induced by aging and chronic denervation impair axonal regeneration following peripheral nerve injury. EMBO Mol. Med. 15:e17907. doi: 10.15252/emmm.202317907, 37860842 PMC10701627

[ref27] Gambarotto L. Russo L. Bresolin S. Persano L. D'Amore R. Ronchi G. . (2025). Schwann cell-specific ablation of Beclin 1 impairs myelination and leads to motor and sensory neuropathy in mice. Adv. Sci. 12:e2308965. doi: 10.1002/advs.202308965, 39680476 PMC11792035

[ref28] Gant K. L. Guest J. D. Palermo A. E. Vedantam A. Jimsheleishvili G. Bunge M. B. . (2022). Phase 1 safety trial of autologous human Schwann cell transplantation in chronic spinal cord injury. J. Neurotrauma 39, 285–299. doi: 10.1089/neu.2020.7590, 33757304 PMC9360180

[ref29] Garcia-Diaz B. Bachelin C. Coulpier F. Gerschenfeld G. Deboux C. Zujovic V. . (2019). Blood vessels guide Schwann cell migration in the adult demyelinated CNS through Eph/ephrin signaling. Acta Neuropathol. 138, 457–476. doi: 10.1007/s00401-019-02011-1, 31011859 PMC6689289

[ref30] Gargareta V. I. Berghoff S. A. Krauter D. Hümmert S. Marshall-Phelps K. L. H. Möbius W. . (2024). Myelinated peripheral axons are more vulnerable to mechanical trauma in a model of enlarged axonal diameters. Glia 72, 1572–1589. doi: 10.1002/glia.24568, 38895764

[ref32] Glenn T. D. Talbot W. S. (2013). Signals regulating myelination in peripheral nerves and the Schwann cell response to injury. Curr. Opin. Neurobiol. 23, 1041–1048. doi: 10.1016/j.conb.2013.06.010, 23896313 PMC3830599

[ref33] Gomzikova M. O. Zhuravleva M. N. Vorobev V. V. Salafutdinov I. Laikov A. V. Kletukhina S. K. . (2019). Angiogenic activity of cytochalasin B-induced membrane vesicles of human mesenchymal stem cells. Cells 9:95. doi: 10.3390/cells9010095, 31906012 PMC7016674

[ref34] Granger N. Blamires H. Franklin R. J. Jeffery N. D. (2012). Autologous olfactory mucosal cell transplants in clinical spinal cord injury: a randomized double-blinded trial in a canine translational model. Brain 135, 3227–3237. doi: 10.1093/brain/aws268, 23169917 PMC3501977

[ref35] Grigoryan T. Stein S. Qi J. J. Wende H. Garratt A. N. Nave K. A. . (2013). Wnt/Rspondin/β-catenin signals control axonal sorting and lineage progression in Schwann cell development. Proc. Natl. Acad. Sci. USA 110, 18174–18179. doi: 10.1073/pnas.1310490110, 24151333 PMC3831430

[ref36] Gross R. E. Mehler M. F. Mabie P. C. Zang Z. Santschi L. Kessler J. A. (1996). Bone morphogenetic proteins promote astroglial lineage commitment by mammalian subventricular zone progenitor cells. Neuron 17, 595–606. doi: 10.1016/s0896-6273(00)80193-2, 8893018

[ref37] Gupta R. Kumari S. Tripathi R. Ambasta R. K. Kumar P. (2023). Unwinding the modalities of necrosome activation and necroptosis machinery in neurological diseases. Ageing Res. Rev. 86:101855. doi: 10.1016/j.arr.2023.101855, 36681250

[ref38] Hajimirzaei P. Tabatabaei F. S. A. Nasibi-Sis H. Razavian R. S. Nasirinezhad F. (2025). Schwann cell transplantation for remyelination, regeneration, tissue sparing, and functional recovery in spinal cord injury: a systematic review and meta-analysis of animal studies. Exp. Neurol. 384:115062. doi: 10.1016/j.expneurol.2024.115062, 39579959

[ref39] Han S. J. Y. Adani V. Farrow E. Parmar B. Chang C. F. Cochran K. . (2026). A dual role for GLI3 signaling in neural crest development. Development 153:dev205209. doi: 10.1242/dev.205209, 41459809 PMC13033380

[ref40] Han S. Gao L. B. Dou X. Q. Wang Z. G. Yang K. K. Li D. D. . (2024). Chiral hydrogel nerve conduit boosts peripheral nerve regeneration via regulation of Schwann cell reprogramming. ACS Nano 18, 28358–28370. doi: 10.1021/acsnano.4c10653, 39403973

[ref42] Heming M. Borsch A. L. Wolbert J. Thomas C. Mausberg A. K. Szepanowski F. . (2025). Multi-omic identification of perineurial hyperplasia and lipid-associated nerve macrophages in human polyneuropathies. Nat. Commun. 16:7872. doi: 10.1038/s41467-025-62964-8 40849297, 40849297 PMC12375038

[ref43] Huang J. H. Chen Y. N. He H. Fu C. H. Xu Z. Y. Lin F. Y. (2022). Schwann cells-derived exosomes promote functional recovery after spinal cord injury by promoting angiogenesis. Front. Cell. Neurosci. 16:1077071. doi: 10.3389/fncel.2022.1077071, 36687521 PMC9846210

[ref44] Huang B. Jiang Y. X. Zhang L. Yang B. Guo Y. J. Yang X. M. . (2023). Low-intensity pulsed ultrasound promotes proliferation and myelinating genes expression of Schwann cells through NRG1/ErbB signaling pathway. Tissue Cell 80:101985. doi: 10.1016/j.tice.2022.101985, 36459840

[ref45] Huang G. J. Li Z. D. Liu X. Z. Guan M. L. Zhou S. L. Zhong X. W. . (2024). DOR activation in mature oligodendrocytes regulates α-ketoglutarate metabolism leading to enhanced remyelination in aged mice. Nat. Neurosci. 27, 2073–2085. doi: 10.1038/s41593-024-01785-2 39266660

[ref46] Huang Y. Y. Ye K. He A. D. Wan S. B. Wu M. B. Hu D. H. . (2024). Dual-layer conduit containing VEGF-A – Transfected Schwann cells promotes peripheral nerve regeneration via angiogenesis. Acta Biomater. 180, 323–336. doi: 10.1016/j.actbio.2024.03.029, 38561075

[ref47] Huang J. S. Zhang G. Y. Li S. R. Li J. N. Wang W. A. Xue J. J. . (2024). Endothelial cell-derived exosomes boost and maintain repair-related phenotypes of Schwann cells via miR199-5p to promote nerve regeneration. J. Nanobiotechnol. 22:81. doi: 10.1186/s12951-023-02289-0 PMC10903063 38419067

[ref48] Ishii A. Furusho M. Bansal R. (2021). Mek/ERK1/2-MAPK and PI3K/Akt/mTOR signaling plays both independent and cooperative roles in Schwann cell differentiation, myelination and dysmyelination. Glia 69, 2429–2446. doi: 10.1002/glia.24049, 34157170 PMC8373720

[ref49] Jacob C. Lötscher P. Engler S. Baggiolini A. Tavares S. V. Brügger V. . (2014). Hdac1 and Hdac2 control the specification of neural crest cells into peripheral glia. J. Neurosci. 34, 6112–6122. doi: 10.1523/Jneurosci.5212-13.2014, 24760871 PMC3996228

[ref50] Jessen K. R. Arthur-Farraj P. (2019). Repair Schwann cell update: adaptive reprogramming, EMT, and stemness in regenerating nerves. Glia 67, 421–437. doi: 10.1002/glia.23532, 30632639

[ref51] Jessen K. R. Mirsky R. (2008). Negative regulation of myelination: relevance for development, injury, and demyelinating disease. Glia 56, 1552–1565. doi: 10.1002/glia.20761, 18803323

[ref52] Jha M. K. Lee Y. Russell K. A. Yang F. Dastgheyb R. M. Deme P. . (2020). Monocarboxylate transporter 1 in Schwann cells contributes to maintenance of sensory nerve myelination during aging. Glia 68, 161–177. doi: 10.1002/glia.23710, 31453649 PMC7054847

[ref53] Kalpachidou T. Kummer K. Handle V. Zimmermann D. Peteinareli M. Quarta S. . (2025). Context dependent role of miR-486 promoting neuroregeneration of primary sensory neurons downstream of interleukin-6 signal transducer. Molecular Therapy Nucleic Acids 36:102670. doi: 10.1016/j.omtn.2025.102670, 40896587 PMC12398936

[ref54] Kantarci H. Elvira P. D. Thottumkara A. P. O'Connell E. M. Iyer M. Donovan L. J. . (2024). Schwann cell-secreted PGE(2) promotes sensory neuron excitability during development. Cell 187, 4690–4712. doi: 10.1016/j.cell.2024.07.033 39142281 PMC11967275

[ref55] Kharazinejad E. Hassanzadeh G. Sahebkar A. Yousefi B. Reza Sameni H. Majidpoor J. . (2023). The comparative effects of Schwann cells and Wharton's jelly mesenchymal stem cells on the AIM2 inflammasome activity in an experimental model of spinal cord injury. Neuroscience 535, 1–12. doi: 10.1016/j.neuroscience.2023.10.011, 37890609

[ref56] Kromer L. F. Cornbrooks C. J. (1985). Transplants of Schwann cell cultures promote axonal regeneration in th e adult mammalian brain. Proc. Natl. Acad. Sci. USA 82, 6330–6334. doi: 10.1073/pnas.82.18.6330, 3862133 PMC391047

[ref57] Kühne B. A. Klose J. Seeger B. Illa M. Muñoz-Torrero D. Koch K. . (2026). Developmental neurotoxicity evaluation of di(2-ethylhexyl) phthalate (DEHP) and three alternative plasticizers in human neurospheres. Environ. Int. 207:110005. doi: 10.1016/j.envint.2025.110005, 41496219

[ref58] Lai X. Liu J. Zou Z. Wang Y. Wang Y. Liu X. . (2021). SOX10 ablation severely impairs the generation of postmigratory neural crest from human pluripotent stem cells. Cell Death Dis. 12:814. doi: 10.1038/s41419-021-04099-4, 34453037 PMC8397771

[ref59] Li Y. Cheng Z. Yu F. Zhang Q. Yu S. Ding F. . (2022). Activin a secreted from peripheral nerve fibroblasts promotes proliferation and migration of Schwann cells. Front. Mol. Neurosci. 15:859349. doi: 10.3389/fnmol.2022.859349, 35875658 PMC9301483

[ref60] Li Y. Field P. M. Raisman G. (1999). Death of oligodendrocytes and microglial phagocytosis of myelin precede immigration of Schwann cells into the spinal cord. J. Neurocytol. 28, 417–427. doi: 10.1023/a:1007026001189, 10739580

[ref61] Li R. Li D. Wu C. Ye L. Wu Y. Yuan Y. . (2020). Nerve growth factor activates autophagy in Schwann cells to enhance myelin debris clearance and to expedite nerve regeneration. Theranostics 10, 1649–1677. doi: 10.7150/thno.40919, 32042328 PMC6993217

[ref62] Li W. Liu G. X. Liang J. Wang X. Song M. Y. Liu X. L. . (2025). The dance between Schwann cells and macrophages during the repair of peripheral nerve injury. Neurosci. Bull. 41, 1448–1462. doi: 10.1007/s12264-025-01427-y, 40483303 PMC12314166

[ref63] Li Y. Y. Luo W. Q. Meng C. K. Shi K. Y. Gu R. Cui S. S. (2024). Exosomes as promising bioactive materials in the treatment of spinal cord injury. Stem Cell Res. Ther. 15:335. doi: 10.1186/s13287-024-03952-5, 39334506 PMC11438208

[ref64] Li P. Xu J. W. Shi Q. S. Wang J. X. Zhang W. X. Zheng L. S. . (2023). Pulse capacitive coupling electric field regulates cell migration, proliferation, polarization, and vascularization to accelerate wound healing. Adv. Wound Care 12, 498–512. doi: 10.1089/wound.2021.0194, 36355602

[ref65] Liu X. Y. Guan J. D. Wu Z. G. Xu L. C. Sun C. (2023). The TGR5 agonist INT-777 promotes peripheral nerve regeneration by activating cAMP-dependent protein kinase a in Schwann cells. Mol. Neurobiol. 60, 1901–1913. doi: 10.1007/s12035-022-03182-x, 36593434

[ref66] Liu B. Xin W. Tan J. R. Zhu R. P. Li T. Wang D. . (2019). Myelin sheath structure and regeneration in peripheral nerve injury repair. Proc. Natl. Acad. Sci. USA 116, 22347–22352. doi: 10.1073/pnas.1910292116, 31611410 PMC6825268

[ref67] Lu M. F. Liu J. P. Xu Y. S. Zuo C. Liu S. C. Zhang W. J. (2025). Application and challenge of Schwann cell transplantation in spinal cord injury and clinical trial. Int. J. Surg. 111, 8284–8300. doi: 10.1097/JS9.0000000000002955, 40844302 PMC12626565

[ref69] Martinez-Morales P. L. del Diez Corral R. Olivera-Martinez I. Quiroga A. C. Das R. M. Barbas J. A. . (2011). FGF and retinoic acid activity gradients control the timing of neural crest cell emigration in the trunk. J. Cell Biol. 194, 489–503. doi: 10.1083/jcb.201011077, 21807879 PMC3153641

[ref71] Mironova Y. A. Dang B. Heo D. Xu Y. K. T. Hsu A. Y. H. von Bernhardi J. E. . (2026). Myelin is repaired by constitutive differentiation of oligodendrocyte progenitors. Science 391:eadu2896. doi: 10.1126/science.adu2896 41570153 PMC12997438

[ref73] Monje P. V. Deng L. X. Xu X. M. (2021). Human Schwann cell transplantation for spinal cord injury: prospects and challenges in translational medicine. Front. Cell. Neurosci. 15:690894. doi: 10.3389/fncel.2021.690894, 34220455 PMC8249939

[ref74] Muscella A. Vetrugno C. Cossa L. G. Marsigliante S. (2020). TGF-beta1 activates RSC96 Schwann cells migration and invasion through MMP-2 and MMP-9 activities. J. Neurochem. 153, 525–538. doi: 10.1111/jnc.14913, 31729763

[ref75] Nair A. Bu J. Rawding P. A. Do S. C. Li H. Hong S. (2021). Cytochalasin B treatment and osmotic pressure enhance the production of extracellular vesicles (EVs) with improved drug loading capacity. Nanomaterials (Basel) 12:3. doi: 10.3390/nano12010003, 35009953 PMC8746776

[ref76] Negro S. Lessi F. Duregotti E. Aretini P. La Ferla M. Franceschi S. . (2017). CXCL12alpha/SDF-1 from perisynaptic Schwann cells promotes regeneration of injured motor axon terminals. EMBO Mol. Med. 9, 1000–1010. doi: 10.15252/emmm.201607257, 28559442 PMC5538331

[ref77] Ngo J. M. Williams J. K. Zhang C. Saleh A. H. Liu X. M. Ma L. . (2025). Extracellular vesicles and cellular homeostasis. Annu. Rev. Biochem. 94, 587–609. doi: 10.1146/annurev-biochem-100924-012717, 40101210

[ref78] Nie X. Y. Yuan T. Y. Yu T. Yun Z. H. Yu T. Liu Q. Y. (2024). Non-stem cell-derived exosomes: a novel therapeutics for neurotrauma. J. Nanobiotechnol. 22:108. doi: 10.1186/s12951-024-02380-0, 38475766 PMC10929230

[ref81] Ou M. Y. Tan P. C. Xie Y. Liu K. Gao Y. M. Yang X. S. . (2022). <article-title update="added">dedifferentiated Schwann cell-derived TGF-β3 is essential for the neural system to promote wound healing. Theranostics 12, 5470–5487. doi: 10.7150/thno.72317, 35910794 PMC9330527

[ref82] Ouyang Y. Yu M. Zhang T. Cheng H. Zuo L. Liu H. . (2025). Single-cell transcriptomic landscape of sciatic nerve after transection injury. J. Neuroinflammation 22:205. doi: 10.1186/s12974-025-03514-3, 40849669 PMC12374453

[ref83] Pardo-Rodríguez B. Baraibar A. M. Manero-Roig I. Luzuriaga J. Salvador-Moya J. Polo Y. . (2025). Functional differentiation of human dental pulp stem cells into neuron-like cells exhibiting electrophysiological activity. Stem Cell Res Ther 16:10. doi: 10.1186/s13287-025-04134-7, 39849603 PMC11756023

[ref84] Parfejevs V. Debbache J. Shakhova O. Schaefer S. M. Glausch M. Wegner M. . (2018). Injury-activated glial cells promote wound healing of the adult skin in mice. Nat. Commun. 9:236. doi: 10.1038/s41467-017-01488-2, 29339718 PMC5770460

[ref85] Park S. Y. Kim G. Liu Y. T. Jung J. W. Lee J. E. Lee J. K. . (2026). Multimodal electroconductive PLGA-based scaffold orchestrates neuroprotection and regeneration following severe spinal cord injury. J. Nanobiotechnol. 24:25. doi: 10.1186/s12951-025-03998-4, 41507980 PMC12794284

[ref86] Parmantier E. Lynn B. Lawson D. Turmaine M. Namini S. S. Chakrabarti L. . (1999). Schwann cell-derived desert hedgehog controls the development of peripheral nerve sheaths. Neuron 23, 713–724. doi: 10.1016/s0896-6273(01)80030-1, 10482238

[ref87] Patel A. A. Kim H. Ramesh R. Marquez A. Faraj M. M. Antikainen H. . (2024). TFEB/3 govern repair Schwann cell generation and function following peripheral nerve injury. J. Neurosci. 44:e0198242024. doi: 10.1523/JNEUROSCI.0198-24.2024, 39054068 PMC11358533

[ref88] Pestronk A. Schmidt R. E. Bucelli R. Sim J. (2023). Schwann cells and myelin in human peripheral nerve: major protein components vary with age, axon size and pathology. Neuropathol. Appl. Neurobiol. 49:e12898. doi: 10.1111/nan.12898, 36868780

[ref89] Piovesana R. Pisano A. Loreti S. Ricordy R. Talora C. Tata A. M. (2022). Notch signal mediates the cross-interaction between M2 muscarinic acetylcholine receptor and neuregulin/ErbB pathway: effects on Schwann cell proliferation. Biomolecules 12:239. doi: 10.3390/biom12020239, 35204740 PMC8961597

[ref90] Podder A. K. Mehrotra P. Lei P. Andreadis S. T. (2025). Enhanced Schwann cell differentiation of skin-derived neural crest-like stem cells through the synergistic action of SOX10 and immobilized NRG1 signaling. Bioengineering & Translational Medicine 10:e70041. doi: 10.1002/btm2.70041, 41244338 PMC12617549

[ref91] Poitelon Y. Lopez-Anido C. Catignas K. Berti C. Palmisano M. Williamson C. . (2016). YAP and TAZ control peripheral myelination and the expression of laminin receptors in Schwann cells. Nat. Neurosci. 19, 879–887. doi: 10.1038/nn.4316, 27273766 PMC4925303

[ref93] Proietti D. Giordani L. De Bardi M. D'Ercole C. Lozanoska-Ochser B. Amadio S. . (2021). Activation of skeletal muscle-resident glial cells upon nerve injury. JCI Insight 6:e143469. doi: 10.1172/jci.insight.143469 33661767 PMC8119188

[ref94] Qian Y. Yan Z. Ye T. Shahin V. Jiang J. Fan C. (2024). Decoding the regulatory role of ATP synthase inhibitory factor 1 (ATPIF1) in Wallerian degeneration and peripheral nerve regeneration. Exploration (Beijing) 4:20230098. doi: 10.1002/EXP.20230098, 39713198 PMC11655313

[ref95] Ren Z. Huang W. Gu X. Zhao L. (2025). AT-rich interaction domain 5A facilitates axon regeneration through docking protein 6 in the peripheral nervous system. Burns Trauma 13:tkaf012. doi: 10.1093/burnst/tkaf012, 40568374 PMC12187523

[ref96] Ren J. Zhu B. Gu G. Zhang W. Li J. Wang H. . (2023). Schwann cell-derived exosomes containing MFG-E8 modify macrophage/microglial polarization for attenuating inflammation via the SOCS3/STAT3 pathway after spinal cord injury. Cell Death Dis. 14:70. doi: 10.1038/s41419-023-05607-4, 36717543 PMC9887051

[ref97] Renthal W. Tochitsky I. Yang L. T. Cheng Y. C. Li E. Kawaguchi R. . (2020). Transcriptional reprogramming of distinct peripheral sensory neuron subtypes after axonal injury. Neuron 108, 128–144. doi: 10.1016/j.neuron.2020.07.026 32810432 PMC7590250

[ref98] Saito D. Takase Y. Murai H. Takahashi Y. (2012). The dorsal aorta initiates a molecular cascade that instructs sympatho-adrenal specification. Science 336, 1578–1581. doi: 10.1126/science.1222369, 22723422

[ref99] Sas A. R. Carbajal K. S. Jerome A. D. Menon R. Yoon C. Kalinski A. L. . (2020). A new neutrophil subset promotes CNS neuron survival and axon regeneration. Nat. Immunol. 21:1496. doi: 10.1038/s41590-020-00813-0, 33106668 PMC7677206

[ref100] Sims T. J. Durgun M. B. Gilmore S. A. (1998). Schwann cell invasion of ventral spinal cord: the effect of irradiation on astrocyte barriers. J. Neuropathol. Exp. Neurol. 57, 866–873. doi: 10.1097/00005072-199809000-00008, 9737550

[ref101] Soós Á. Szőcs E. Halasy V. Gecse Z. Mógor F. Jurenka C. . (2025). Cecal growth factors promote enteric neurosphere formation and hindgut colonization in the avian model. Front. Cell Dev. Biol. 13:1681844. doi: 10.3389/fcell.2025.1681844, 41488003 PMC12756466

[ref102] Stavely R. Hotta R. Picard N. Rahman A. A. Pan W. K. Bhave S. . (2022). Schwann cells in the subcutaneous adipose tissue have neurogenic potential and can be used for regenerative therapies. Sci. Transl. Med. 14:eabl8753. doi: 10.1126/scitranslmed.abl8753, 35613280 PMC9745588

[ref103] Stierli S. Sommer L. (2022). Schwann cell precursors: a hub of neural crest development. EMBO J. 41:e111955. doi: 10.15252/embj.2022111955, 35894449 PMC9434098

[ref104] Stundl J. Desingu Rajan A. R. Urrutia H. A. Leyhr J. Stundlova J. Solovieva T. . (2025). Acquisition of neural crest promoted thyroid evolution from chordate endostyle. Sci. Adv. 11:eadv2657. doi: 10.1126/sciadv.adv2657, 40768591 PMC12327467

[ref105] Su W. F. He X. W. Lin Z. H. Xu J. H. Shangguan J. H. Wei Z. Y. . (2025). Activation of P2X7R inhibits proliferation and promotes the migration and differentiation of Schwann cells. Mol. Neurobiol. 62, 3067–3081. doi: 10.1007/s12035-024-04460-6, 39225968

[ref106] Suzuki T. Kadoya K. Endo T. Iwasaki N. (2023). Molecular and regenerative characterization of repair and non-repair Schwann cells. Cell. Mol. Neurobiol. 43, 2165–2178. doi: 10.1007/s10571-022-01295-4, 36222946 PMC11412190

[ref107] Tseropoulos G. Mehrotra P. Podder A. K. Wilson E. Zhang Y. Wang J. . (2024). Immobilized NRG1 accelerates neural crest like cell differentiation toward functional Schwann cells through sustained Erk1/2 activation and YAP/TAZ nuclear translocation. Adv. Sci. 11:e2402607. doi: 10.1002/advs.202402607, 38952126 PMC11633358

[ref110] Wagstaff L. J. Gomez-Sanchez J. A. Fazal S. V. Otto G. W. Kilpatrick A. M. Michael K. . (2021). Failures of nerve regeneration caused by aging or chronic denervation are rescued by restoring Schwann cell c-Jun. eLife 10:e62232. doi: 10.7554/eLife.62232, 33475496 PMC7819709

[ref111] Wan B. Li C. Wang M. Kong F. Ding Q. Zhang C. . (2021). GIT1 protects traumatically injured spinal cord by prompting microvascular endothelial cells to clear myelin debris. Aging 13, 7067–7083. doi: 10.18632/aging.202560, 33621952 PMC7993661

[ref112] Wan T. Zhang F. S. Qin M. Y. Jiang H. R. Zhang M. Qu Y. . (2024). Growth factors: bioactive macromolecular drugs for peripheral nerve injury treatment – Molecular mechanisms and delivery platforms. Biomed. Pharmacother. 170, 116024–116015. doi: 10.1016/j.biopha.2023.116024, 38113623

[ref113] Wang Q. Chen F. Y. Ling Z. M. Su W. F. Zhao Y. Y. Chen G. . (2022). The effect of Schwann cells/Schwann cell-like cells on cell therapy for peripheral neuropathy. Front. Cell. Neurosci. 16:836931. doi: 10.3389/fncel.2022.836931, 35350167 PMC8957843

[ref114] Wang P. Wang R. Huo Y. Peng Y. Fu C. Hu Y. . (2025). Partial reprogramming in senescent Schwann cells enhances peripheral nerve regeneration via restoration of stress granule homeostasis. Adv. Sci. 12:e11019. doi: 10.1002/advs.202511019, 40899516 PMC12667534

[ref115] Wang Y. Xiong Z. Qiao Y. Zhang Q. Zhou G. Zhou C. . (2024). Acetyl-11-keto-beta-boswellic acid modulates macrophage polarization and Schwann cell migration to accelerate spinal cord injury repair in rats. CNS Neurosci. Ther. 30:e14642. doi: 10.1111/cns.14642, 38430464 PMC10908365

[ref116] Wang H. K. Zhang P. Lu P. J. Cai X. D. Wang G. Xu X. . (2023). Neural tissue-engineered prevascularization enhances peripheral neuroregeneration via rapid vascular inosculation. Mater. Today Bio 21:100718. doi: 10.1016/j.mtbio.2023.100718 PMC10339252 37455820

[ref117] Wang Y. X. Zhang F. C. Zhang Y. S. Shan Q. Liu W. Zhang F. Y. . (2021). Betacellulin regulates peripheral nerve regeneration by affecting Schwann cell migration and axon elongation. Mol. Med. 27:27. doi: 10.1186/s10020-021-00292-5 33794764 PMC8015203

[ref119] Wei G. H. Chen C. L. Li X. Wang H. Y. Li Z. Q. Gou X. . (2025). In situ piezoelectricity induces M2 polarization of macrophages to regulate Schwann cells for alleviating neuropathic pain of CCI rats. Biomater. Adv. 174:214319. doi: 10.1016/j.bioadv.2025.214319, 40245815

[ref120] Wei J. H. Su W. F. Zhao Y. Y. Wei Z. Y. Hua Y. C. Xue P. . (2022). Maresin 1 promotes nerve regeneration and alleviates neuropathic pain after nerve injury. J. Neuroinflammation 19:32. doi: 10.1186/s12974-022-02405-1 35109876 PMC8809034

[ref121] Weiss T. Taschner-Mandl S. Janker L. Bileck A. Rifatbegovic F. Kromp F. . (2021). Schwann cell plasticity regulates neuroblastic tumor cell differentiation via epidermal growth factor-like protein 8. Nat. Commun. 12:1624. doi: 10.1038/s41467-021-21859-0, 33712610 PMC7954855

[ref122] Wilcox M. B. Laranjeira S. G. Eriksson T. M. Jessen K. R. Mirsky R. Quick T. J. . (2020). Characterising cellular and molecular features of human peripheral nerve degeneration. Acta Neuropathol. Commun. 8:51. doi: 10.1186/s40478-020-00921-w, 32303273 PMC7164159

[ref123] Wilson E. R. Nunes G. D. Shen S. C. Moore S. Gawron J. Maxwell J. . (2024). Loss of prohibitin 2 in Schwann cells dysregulates key transcription factors controlling developmental myelination. Glia 72, 2247–2267. doi: 10.1002/glia.24610, 39215540 PMC11577967

[ref124] Woodhoo A. Alonso M. B. Droggiti A. Turmaine M. D'Antonio M. Parkinson D. B. . (2009). Notch controls embryonic Schwann cell differentiation, postnatal myelination and adult plasticity. Nat. Neurosci. 12, 839–847. doi: 10.1038/nn.2323 19525946 PMC2782951

[ref125] Woodhoo A. Sommer L. (2008). Development of the Schwann cell lineage: from the neural crest to the myelinated nerve. Glia 56, 1481–1490. doi: 10.1002/glia.20723, 18803317

[ref126] Wu Z. Ding H. Chen Y. Huang C. Chen X. Hu H. . (2023). Motor neurons transplantation alleviates neurofibrogenesis during chronic degeneration by reversibly regulating Schwann cells epithelial-mesenchymal transition. Exp. Neurol. 359:114272. doi: 10.1016/j.expneurol.2022.114272, 36370841

[ref127] Wu L. M. Wang J. Conidi A. Zhao C. Wang H. Ford Z. . (2016). Zeb2 recruits HDAC-NuRD to inhibit notch and controls Schwann cell differentiation and remyelination. Nat. Neurosci. 19, 1060–1072. doi: 10.1038/nn.4322, 27294509 PMC4961522

[ref128] Wu L. Zhu Y. Meng Q. (2026). Exosome-loaded hydrogel systems for spinal cord injury repair: mechanisms, advancements, and future directions. J. Mater. Sci. Mater. Med. 37:35. doi: 10.1007/s10856-025-06931-1, 41636885 PMC12886294

[ref129] Xu X. Liang Z. Lin Y. Rao J. Lin F. Yang Z. . (2022). Comparing the efficacy and safety of cell transplantation for spinal cord injury: a systematic review and Bayesian network Meta-analysis. Front. Cell. Neurosci. 16:860131. doi: 10.3389/fncel.2022.860131, 35444516 PMC9013778

[ref130] Xu X. Song L. Li Y. Guo J. Huang S. Du S. . (2023). Neurotrophin-3 promotes peripheral nerve regeneration by maintaining a repair state of Schwann cells after chronic denervation via the TrkC/ERK/c-Jun pathway. J. Transl. Med. 21:733. doi: 10.1186/s12967-023-04609-2, 37848983 PMC10583391

[ref131] Xu B. Zhou Z. Fang J. Wang J. Tao K. Liu J. . (2023). Exosomes derived from schwann cells alleviate mitochondrial dysfunction and necroptosis after spinal cord injury via AMPK signaling pathway-mediated mitophagy. Free Radic. Biol. Med. 208, 319–333. doi: 10.1016/j.freeradbiomed.2023.08.026, 37640169

[ref132] Yadav A. Huang T. C. Chen S. H. Ramasamy T. S. Hsueh Y. Y. Lin S. P. . (2021). Sodium phenylbutyrate inhibits Schwann cell inflammation via HDAC and NFκB to promote axonal regeneration and remyelination. J. Neuroinflammation 18:238. doi: 10.1186/s12974-021-02273-1, 34656124 PMC8520633

[ref133] Yamauchi J. Miyamoto Y. Chan J. R. Tanoue A. (2008). ErbB2 directly activates the exchange factor Dock7 to promote Schwann cell migration. J. Cell Biol. 181, 351–365. doi: 10.1083/jcb.200709033, 18426980 PMC2315680

[ref134] Yang S. H. Wang C. Zhu J. J. Lu C. F. Li H. T. Chen F. Y. . (2020). Self-assembling peptide hydrogels functionalized with LN- and BDNF- mimicking epitopes synergistically enhance peripheral nerve regeneration. Theranostics 10, 8227–8249. doi: 10.7150/thno.44276, 32724468 PMC7381722

[ref135] Ye K. Li Z. T. Y. Yin Y. H. Zhou J. Li D. J. Gan Y. . (2023). LIPUS-SCs-Exo promotes peripheral nerve regeneration in cavernous nerve crush injury-induced ED rats via PI3K/Akt/FoxO signaling pathway. CNS Neurosci. Ther. 29, 3239–3258. doi: 10.1111/cns.14256, 37157936 PMC10580359

[ref136] Yim A. K. Y. Wang P. L. Bermingham J. R. Jr. Hackett A. Strickland A. Miller T. M. . (2022). Disentangling glial diversity in peripheral nerves at single-nuclei resolution. Nat. Neurosci. 25, 238–251. doi: 10.1038/s41593-021-01005-1, 35115729 PMC9060899

[ref137] Yu M. M. Shen M. Chen D. Y. Li Y. Zhou Q. Deng C. Y. . (2024). Chitosan/PLGA-based tissue engineered nerve grafts with SKP-SC-EVs enhance sciatic nerve regeneration in dogs through miR-30b-5p-mediated regulation of axon growth. Bioact. Mater. 40, 378–395. doi: 10.1016/j.bioactmat.2024.06.011 38978801 PMC11228890

[ref138] Yum Y. Park S. Nam Y. H. Yoon J. Song Kim H. J. . (2024). Therapeutic effect of Schwann cell-like cells differentiated from human tonsil-derived mesenchymal stem cells on diabetic neuropathy in db/db mice. Tissue Eng. Regen. Med. 21, 761–776. doi: 10.1007/s13770-024-00638-0 38619758 PMC11187028

[ref139] Zhai Y. H. Guan X. H. Lu C. R. Sun R. X. Qian Y. Li Y. . (2026). Injectable chitosan-based hydrogel via in situ gelation modulates the inflammatory microenvironment and facilitates minimally invasive repair of peripheral nerve injury. Mater. Today Bio 37:102814. doi: 10.1016/j.mtbio.2026.102814, 41624516 PMC12859466

[ref140] Zhang Q. Y. Chen Y. X. Huang W. Zhou J. Q. Yang D. W. (2025). Melittin promotes the proliferation of Schwann cells in hyperglycemic environment by up-regulating the Crabp2/Wnt/β-catenin signaling pathway. Mol. Med. Rep. 31:13371. doi: 10.3892/mmr.2024.13371 PMC11529206 39450531

[ref141] Zhang Q. Du Y. J. Xu D. Y. Zhang H. M. Li Y. Y. Li L. X. . (2025). Sonic hedgehog promotes Schwann cell proliferation through PI3K/AKT/ cyclin E1 pathway. Tissue Cell 95:102858. doi: 10.1016/j.tice.2025.102858 40106859

[ref142] Zhang Y. Li X. Ciric B. Curtis M. T. Chen W. J. Rostami A. . (2020). A dual effect of ursolic acid to the treatment of multiple sclerosis through both immunomodulation and direct remyelination. Proc. Natl. Acad. Sci. USA 117, 9082–9093. doi: 10.1073/pnas.2000208117, 32253301 PMC7183235

[ref143] Zhang H. M. Wang S. J. Zhang Q. Du X. Y. Xu D. Y. Wen J. K. . (2025). Indole-3-propionic acid promotes Schwann cell proliferation following peripheral nerve injury by activating the PI3K/AKT pathway. Neurotherapeutics 22:e00578. doi: 10.1016/j.neurot.2025.e00578, 40148157 PMC12418468

[ref144] Zhang Z. Yu B. Gu Y. Zhou S. Qian T. Wang Y. . (2016). Fibroblast-derived tenascin-C promotes Schwann cell migration through β1-integrin dependent pathway during peripheral nerve regeneration. Glia 64, 374–385. doi: 10.1002/glia.22934, 26497118

[ref145] Zheng B. H. Tuszynski M. H. (2023). Regulation of axonal regeneration after mammalian spinal cord injury. Nat. Rev. Mol. Cell Biol. 24, 396–413. doi: 10.1038/s41580-022-00562-y, 36604586

[ref146] Zhou H. L. Zhang X. J. Zhang M. Y. Yan Z. J. Xu Z. M. Xu R. X. (2016). Transplantation of human amniotic mesenchymal stem cells promotes functional recovery in a rat model of traumatic spinal cord injury. Neurochem. Res. 41, 2708–2718. doi: 10.1007/s11064-016-1987-9, 27351200

[ref147] Zhu B. Gu G. Ren J. Song X. Li J. Wang C. . (2023). Schwann cell-derived exosomes and methylprednisolone composite patch for spinal cord injury repair. ACS Nano 17, 22928–22943. doi: 10.1021/acsnano.3c08046, 37948097

